# CD4 ^+^ differentiated T regulatory cells is modified by physical fitness and visceral adipose tissue in young adults—A cross‐sectional study

**DOI:** 10.14814/phy2.70470

**Published:** 2025-08-25

**Authors:** Camila S. Padilha, Tiago Olean‐Oliveira, Caique Figueiredo, Vanessa R. Dos Santos, Gilson P. Dorneles, José Procópio Jabur Ribeiro, Rafael Deminice, Karsten Krüger, José C. Rosa‐Neto, Fábio S. Lira

**Affiliations:** ^1^ Centre for Healthy Ageing, Biology of Ageing Laboratory Centenary Institute of Cancer Medicine and Cell Biology, Royal Prince Alfred Hospital Sydney New South Wales Australia; ^2^ Faculty of Medicine and Health, Charles Perkins Centre University of Sydney Sydney New South Wales Australia; ^3^ School of Sport, Exercise and Rehabilitation Sciences University of Technology Sydney Sydney New South Wales Australia; ^4^ Exercise and Immunometabolism Research Group, Post‐Graduation Program in Movement Sciences, Department of Physical Education São Paulo State University (UNESP) Presidente Prudente SP Brazil; ^5^ Immunometabolism Research Group, Department of Cell Biology and Development, Institute of Biomedical Science University of São Paulo São Paulo Brazil; ^6^ Musculoskeletal System Assessment Laboratory, Post‐Graduation Program in Movement Sciences, Department of Physical Education Universidade Estadual Paulista (UNESP) Presidente Prudente Brazil; ^7^ Cellular and Molecular Immunology Laboratory Federal University of Healthy Science of Porto Alegre Porto Alegre RS Brazil; ^8^ Hospital Moinhos de Vento Porto Alegre RS Brazil; ^9^ Laboratory of Biochemistry Exercise, Department of Physical Education, Faculty of Physical Education and Sport State University of Londrina Londrina Brazil; ^10^ Department of Exercise Physiology and Sports Therapy, Institute of Sports Science Justus Liebig University Giessen Giessen Germany; ^11^ Research Center for Sport and Physical Activity, Faculty of Sports Science and Physical Education The University of Coimbra Coimbra Portugal

**Keywords:** central obesity, fitness status, immunometabolism, lymphocytes

## Abstract

Central adiposity and poor cardiorespiratory fitness are modifiable risk factors for various diseases. This study investigated their impact on CD4^+^ differentiated T regulatory (Treg) cell responses. Thirty‐eight young adults were classified into high cardiorespiratory fitness/low visceral adipose tissue (High V̇O_2_–Low VAT, *n* = 20) and low cardiorespiratory fitness/high VAT (Low V̇O_2_–High VAT, *n* = 18). Body composition was assessed using DXA and ultrasound, while cardiorespiratory fitness and physical activity were measured via treadmill testing and accelerometry. CD4^+^ cells were cultured in Treg differentiation medium with 2 ng/mL TGF‐β, with or without 100 nM rapamycin or 50 nM Torin‐1, for 96 h. Differentiated Treg from Low V̇O_2_–High VAT participants exhibited no significant changes in IL‐10 or IL‐6 production with rapamycin or Torin‐1. Conversely, differentiated Treg from High V̇O_2_–Low VAT participants showed significantly lower IL‐10 production with rapamycin (*p* < 0.001, adjusted *p* < 0.001) and Torin‐1 (*p* < 0.001, adjusted *p* < 0.001). These findings indicate that low cardiorespiratory fitness and high VAT contribute to an altered inflammatory response, influencing peripheral blood mononuclear cell immunophenotypes and exhaustion markers. Furthermore, mTORC1 and mTORC2 inhibition modulate cytokine production, emphasizing the role of metabolic status in immune regulation.

## Graphical abstract

High cardiorespiratory fitness and low visceral adiposity modulate CD4^+^ T regulatory cells. Fit participants shows be more responsible to mTORC1/2 inhibition via rapamycin or Torin‐1indicating a fitness‐ and adiposity‐dependent immunometabolic regulation.

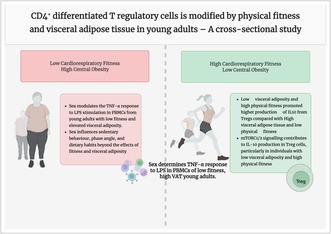

## 1 INTRODUCTION

Central obesity and low cardiorespiratory fitness are strongly associated with several chronic diseases linked to metabolic disturbances leading to increasing morbidity and mortality (Dhokte & Czaja, 2024; Myers et al., 2002; Nazare et al., 2012). Hypertrophic visceral adipose tissue (VAT) is an immunoendocrine and highly active metabolic organ composed not only of adipocytes but also of immune cells (i.e., M1‐like macrophages and CD4^+^ T cells) that produce and release pro‐inflammatory cytokines, which lead to tissue and systemic metabolic stress (Ohlson et al., 1985; Wang et al., 2013). T cells are a critical component of adaptive immunity and are highly responsive to chronic inflammatory and metabolic stress conditions, which reduce the proportion of type 2 T helper (Th2) and regulatory T (Treg) cells and increase the number of pro‐inflammatory populations, such as T helper 1 (Th1), T helper 17 (Th17), and cytotoxic CD8^+^ T cells. In this regard, Treg cells are critical for controlling inflammation and systemic metabolism via interleukin‐10 and transforming growth factor‐beta (IL‐10 and TGF‐β) secretion and suppression of pro‐inflammatory cells (Li et al., 2020). Although chronic inflammation diminishes both the number and function of Treg cells, evidence suggests that regular physical exercise and reduced body fat are closely associated with restoring Treg function and mitigating chronic low‐grade inflammation (Bedke et al., 2019). However, the metabolic programming that underlies these adaptations remains an area of active investigation.

Recently, Rosa‐Neto et al. (2022) suggested that energy‐sensing pathways, such as AMPK (5′ AMP‐activated protein kinase), PPAR‐γ (peroxisome proliferator‐activated receptor gamma), HIF‐1α (hypoxia‐inducible factor 1‐alpha), and mTORC, may be involved in the metabolic programming of immune cells. The mechanistic target of rapamycin (mTOR) signaling pathway, comprising complex 1 (mTORC1) and complex 2 (mTORC2), plays a crucial role in regulating T‐cell metabolism, including the function and maintenance of Treg cells (Zeng & Chi, 2017). mTORC1 activation promotes anabolic processes, whereas mTORC2 regulates cytoskeletal organization and survival signaling—both essential for Treg differentiation and suppressive function (Shi & Chi, 2019; Zeng & Chi, 2017). Studies suggest that mTORC1 supports the metabolic demands of Tregs primarily through glycolysis, while mTORC2 helps maintain their stability (Kempkes et al., 2019; Wang et al., 2020; Zeng & Chi, 2017). However, the precise mechanisms by which these complexes influence Treg responses in inflammatory conditions remain unclear. While mTORC1 and mTORC2 activation modulate Treg cell function, it is uncertain whether inflammatory changes driven by variations in physical fitness and visceral fat alter this dependency. Research on this topic, particularly in humans, remains limited, and the influence—or potential loss of influence—of central obesity and insufficient cardiorespiratory fitness, as well as sex‐related differences, has yet to be fully explored.

Therefore, this study aimed to test the hypothesis that central obesity and cardiorespiratory fitness influence the anti‐inflammatory function of CD4^+^ T cells differentiated into Tregs in an mTOR pathway‐dependent manner in young adult males and females. Additionally, “global‐level” experiments, including peripheral blood mononuclear cells (PBMC) cultures and stimulated whole blood assays, will be conducted to examine the inflammatory response arising from the interaction between Treg cells and other immune cell populations. Establishing the protective effects of increased physical fitness and reduced central obesity on inflammation—particularly in Treg cells—may provide valuable insights for developing strategies to mitigate chronic inflammatory and metabolic disorders associated with obesity and sedentary lifestyles.

## 2 METHODS

### 2.1 Study design and ethics approval

This study presents a cross‐sectional design following the Strengthening The Reporting of Observational Studies in Epidemiology (STROBE) checklist. Young adults who self‐reported male and female sex assigned at birth (18–35 years) were recruited to participate in this study after the flattening of mortality numbers induced by the COVID‐19 pandemic. The participants were included if they tested negative for anti‐SARS‐CoV‐2 (IgG and IgM antibodies), did not present any health disorders (e.g., cardiorespiratory and osteoarticular diseases), and did not use any ergogenic substance or medication for at least 6 months before the study. All female participants were evaluated during the 7th to 12th day of the menstrual cycle and were not undergoing in contraceptive methods. Written informed consent was obtained from all the participants. This study was approved by the local Research Ethics Committee of the São Paulo State University “Júlio de Mesquita Filho” and duly registered in the Brazil Platform, a national electronic system created by the Federal Government to systematize the receipt of research projects involving human beings in Ethics Committees throughout the country (CAAE: 26011919.0.0000.5402). All experiments were conducted according to the 2013 Revision of the Declaration of Helsinki.

### 2.2 Participants' sample size and experimental protocols

The sample size and statistical power were calculated considering a large effect size (0.60 Cohens' *d* and *β* − 1 = 0.90) using the G*Power 3.1 software. A total of 24 individuals was indicated to be sufficient to detect the prescribed effects within this study protocol. We included 38 young adults (men = 29 and women = 9), who were classified as either high oxygen uptake (V̇O_2_)peak–low VAT (*n* = 20) or low V̇O_2_peak–high VAT (*n* = 18).

### 2.3 Maximal incremental test

Participants were submitted to a maximal incremental test on a treadmill (Inbrasport CG‐04; Embramed, Porto Alegre, Brazil) consisting of 2‐min stages until exhaustion. The physical activity level was classified initially through the International Physical Activity Questionnaire (IPAQ) to determine the initial workload of the incremental test and, subsequently, confirmed by the maximal oxygen uptake. The warm‐up workload was set at 5.0 km/h for 5 min. The initial workload was 6.0 km/h for participants not regularly engaged in physical activity and 8.0 km/h for highly active participants, with an increment of 1.0 km/h at each subsequent stage (Caputo & Denadai, 2008). The V̇O_2_ was assessed by a breath‐by‐breath gas analyzer (Quark PFT; Cosmed®, Rome, Italy). The variables measured during the test were as follows: (1) V̇O_2_peak, assumed as the highest 30‐s mean observed during the incremental test; (2) maximal heart rate (HR_Max_) (Polar S810i, Polar Electro Oy®, Kempele, Finland); and (3) maximum power output (W_Max_). The exhaustion criteria were confirmed by the following variables: gas exchange ratio >1.1, HR_Max_ > 90% of the maximum expected for age, and rating of perceived exertion (RPE) >18. It was considered low‐cardiorespiratory fitness level values of V̇O_2_peak between 30 and 45 mL kg^−1^ min^−1^ and high cardiorespiratory fitness level values above 55 mL kg^−1^ min^−1^ according to previous research performed in our laboratory (Antunes et al., 2020; Dorneles et al., 2021).

### 2.4 Measures of physical activity level

The physical activity was measured using an accelerometer (GT3X; ActiGraph LLC, Pensacola, FL, USA). The participants used the accelerometer for 7 days (a minimum of 4 days for at least 10 h a day to be included in the analysis). We defined non‐use time intervals of at least 60 consecutive minutes of zero count, with an activity interruption allowance of 0–100 counts per minute with a maximum duration of two consecutive minutes. The values of counts per minute were calculated as the sum of the total activity count divided by the number of valid days. Sedentary time was delineated as values <100 counts per minute and moderate‐vigorous physical activity as >2020 counts per minute (Troiano et al., 2008). Data were analyzed using the ActLife software (version 6.9.2, Pensacola, FL, USA).

### 2.5 Anthropometric and body composition assessment

The body weight was evaluated using an electronic scale (Filizola PL50 Ltd., Brazil), and the height was measured using a fixed stadiometer with an accuracy of 0.1 cm. The waist circumference (WC) was measured using a measuring tape. The body composition (lean soft mass, fat mass, and bone mineral content) was evaluated by dual‐energy x‐ray absorptiometry (DXA; Lunar DPX‐NT scanner, General Electric Healthcare); also, fat mass (%), free fat mass (%), android and gynoid fat mass (%), and the ratio between the android and gynoid (A/G ratio) were obtained. Additionally, the VAT and subcutaneous (SAT) adipose tissue were evaluated using an ultrasound device (TOSHIBA‐Eccocee, convex transducer of 3.7 MHz, Tokyo, Japan) operated by a physician from an institution specialized in imaging diagnosis (President Prudente Institute of Radiology, Brazil). The parameters and methods for determining VAT were according to Ribeiro‐Filho et al. (2003). Finally, the phase angle was assessed using bioelectrical impedance as arc‐tangent (Xc/R) × 180°/π (Lukaski et al., 2017).

### 2.6 Dietary intake analysis

We analyzed the participants' habitual food consumption using a self‐report protocol on two weekdays and one weekend day. Participants were instructed by a nutritionist as to how to complete the dietary questionnaires. All food intakes were analyzed for total kilocalorie and macronutrient intakes averaged for the 3 days using the software (Software—Dietpro version 5.8) according to the database of Brazilian food composition table (TACO) to calculate dietary intake.

### 2.7 Blood sample and analysis

It was collected approximately 20 mL of blood through a peripheral puncture of a forearm vein, which followed two distinct procedures: (1) immediately allocated to vacutainer tubes with EDTA or an anticoagulant gel for plasma or serum separation, and (2) it was kept at room temperature for cell culture procedures. The blood was collected after a 12‐h fasting period at the same time of the day (between 7:00 a.m. and 9 a.m.) to mitigate the effects of the circadian cycle. The blood samples designed for plasma or serum separation were centrifuged at 2500 x *g* for 15 min at 4°C, and the plasma and serum were stored at −80°C until further colorimetric and enzyme‐linked immunosorbent assay (ELISA) analyses. Glucose concentrations were analyzed using colorimetric kits (Ref: 84‐1/500; Labtest, Brazil), and insulin using ELISA commercial kits (Ref: 2425‐300, Monobind Inc., USA). Homeostasis model assessment of insulin resistance (HOMA‐IR) was calculated using the equation: HOMA‐IR = (glucose [mmol/L] × insulin [μIU/mL]/22.5) (Matthews et al., 1985). Triacylglycerol (TAG) (Ref: 87‐2/250; Labtest®, Brazil) and total cholesterol were analyzed by commercial colorimetric kits (Ref: 76‐2/250; Labtest®, Brazil).

The serum concentrations of leptin and adiponectin were analyzed by ELISA immunoassay method according to the manufacturer's instructions using an assay ELISA with commercial kits (Duoset R&D System, Minneapolis, USA). The concentration of cytokines presents in the supernatants from stimulated whole blood (IL‐6 and TNF‐α), PBMC cultures (IL‐6 and TNF‐α), cultured CD4^+^ cells (IL‐10, IL‐17, IL‐6 and TNF‐α), and cultured CD4^+^ differentiated Treg (IL‐10 and TNF‐α) were determined by ELISA with commercial kits (Duoset R&D System, Minneapolis, USA) with an intra‐assay coefficient of variation of 1.5% for TNF‐α (Ref: DY210), 1.3% for IL‐6 (Ref: DY206), 2.1% for IL‐10 (Ref: DY217B).

### 2.8 Whole blood stimulated with endotoxin

Was used a protocol similar to that described by Barry et al. (2018) for the whole blood stimulated ex vivo assay. Approximately 1 mL of blood from tubes containing K3‐EDTA was diluted 1:10 in serum‐free RPMI‐1040 medium (Ref: MFCD00217820; Sigma‐Aldrich Co. LLC) containing antibiotics penicillin (100 U/mL) and streptomycin (0.1 mg/mL) (Ref: MFCD00130520, Sigma‐Aldrich Co. LLC). Diluted whole blood was plated in 24‐well culture plates (540 μL) and incubated in the presence or absence of lipopolysaccharide (LPS) (Escherichia coli, type: 0111: B4; Ref: 93572‐42‐0, Sigma, St. Louis, MO) at the final concentration of 10 ng/mL for 6 h at 37°C in 5% CO_2_. After this period, the supernatant was collected and stored at −80°C for further analysis.

### 2.9 Peripheral blood mononuclear cells culture isolation and mitogen stimulation

It was added approximately 15 mL of blood to Histopaque®‐1077 (Ref: 10771, Sigma‐Aldrich Co. LLC) (1:1) for PBMC isolation and then centrifuged at 400 × *g* for 30 min at room temperature. The PBMC were washed with phosphate‐buffered saline (PBS) and resuspended in 1 mL of enriched medium RPMI for stimulation with mitogens or cryopreserved for analysis of flow cytometry (Gonçalves and Sobral, 2020). A total of 1.0 × 10^6^ PBMC/mL were incubated for 24 h at 37°C and 5% CO_2_ in cell culture medium (RPMI‐1040, Sigma‐Aldrich Co. LLC) enriched with glutamine (2 mM), HEPES (20 mM), 10% fetal bovine serum, and antibiotics penicillin (100 U/mL) and streptomycin (0.1 mg/mL) in 24‐well plates (Kasvi, PR/Brazil). PBMCs were cultured in the absence or presence of LPS (10 ng/mL) (*Escherichia coli*, type: 0111: B4; Ref: 93572‐42‐0, Sigma, St. Louis, MO) to measure innate inflammatory response, or with Phorbol 12‐Myristate 13‐Acetate (PMA) (50 ng/mL) (Ref: 16561‐29‐8; Sigma, St. Louis, MO) plus ionomycin (1 μg/mL) (Ref: 56092‐82‐1; Sigma, St. Louis, MO) to verify adaptive cytokine production. After 24 h, supernatants were collected and stored at −80°C for further cytokine analysis.

### 2.10 Flow cytometry

The PBMC were thawed by diluting them in 5 ‐mL pre‐warmed complete RPMI‐1640 medium (Sigma‐Aldrich, R8758) containing 5% FBS and spun at 2500 x *g*  for 5 min. Supernatants were carefully removed, and cells were resuspended in RPMI‐1640 medium. The viability of cells (>98%) was examined using trypan‐blue staining (Gibco, Grand Island, New York, USA). Briefly, 2 × 10^5^ PBMCs were stained with monoclonal antibodies (all anti‐human) conjugated with specific fluorochromes: CD4 FITC (Clone OKT‐4), CD8 Pe (Clone RPA‐T8), CD25 Pe (Clone BC 96), CD127 Percp‐Cy5.5 (Clone eBioRDR5), CD28 Percp‐Cy5.5 (Clone CD28.2), PD‐1 APC (Clone MIH 4), CD14 FITC (Clone 61D3), CD16 Pe (Clone eBioCB16), and HLA‐DR Percp‐Cy7 (Clone G46‐6) (Invitrogen, USA). Cell phenotype was acquired using CELLQuest Pro Software (BD Bioscience, USA) on a FACSCalibur flow cytometer (BD Bioscience, USA). A minimum of 20 000 events/tubes were acquired, and lymphocytes were identified and gated according to each forward scatter (FSC) and side scatter (SSC) profile. The mean fluorescence intensity (MFI) of CD28 and PD‐1 was analyzed in CD4^+^ and CD8^+^ T‐cell subpopulations, and HLA‐DR expression was evaluated in CD14^+^CD16^−^ and CD14^+^CD16^+^ monocyte subsets. The Treg phenotype was defined as CD4^+^CD25^high^CD127^low^ according to Liu et al. (2006). For flow cytometry data, measurements from the low V̇O_2_peak–high VAT group were set as relative to the high V̇O_2_peak–low VAT (100%).

### 2.11 CD4 ^+^ T cells isolated from whole blood and differentiation into Treg cells

CD4^+^ T cells were isolated from whole blood using negative selection by the EasySep Kit (Ref: 19662; STEMCELL Technologies, USA) following the manufacturer's instructions. Isolated CD4^+^ T cells were seeded at a density of 2.0 × 10^5^ per well in 24‐well plates (Kasvi, PR / Brazil) with Treg differentiation medium from the commercial kit CellXVivo Human Treg Cell Differentiation Kit (Ref: CDK006; R&D Systems, Inc., Minneapolis, USA), according to the manufacturer's instructions, in the presence of 2 ng/mL of TGF‐β (Ref: 78067; STEMCELL Technologies, USA). To inhibit mTORC1 or mTORC2, CD4^+^ cultured in Treg differentiation medium were seeded in the presence of 100 nM of rapamycin (Ref: 73362; STEMCELL Technologies, USA) or 50 nM of Torin‐1 (Ref: 73492; STEMCELL Technologies, USA), respectively, and incubated for 96 h at 37°C and 5% CO_2_. After 96 h, supernatants were collected and stored at −80°C for further cytokine analysis.

### 2.12 Statistical analysis

The Shapiro–Wilk test was used to verify the data distribution. Descriptive statistics were presented as mean ± standard deviation (SD) for parametric variables, and median and interquartile range (IQR) for non‐parametric variables. The analyses were not stratified by sex due to insufficient statistical power. Therefore, sex, percentage of body fat, BMI, waist circumference, and age were adopted as a covariates based on our study design. A two‐way analysis of covariance (ANCOVA) followed by Tukey's post hoc test was performed. In variables where sphericity was violated as indicated by Mauchly's test, the analyses were adjusted using a Greenhouse–Geisser correction. Statistical significance was set at *p* < 0.05, and the data were analyzed using the Statistical Package for Social Sciences 22.0 (SPSS Inc., Chicago, IL, USA).

## 3 RESULTS

### 3.1 Physical activity according to cardiorespiratory fitness and VAT in young adult males and females

Young adult males and females were classified as High V̇O_2_–Low VAT (male: 76.19% and female: 23.81%; V̇O_2_peak (mL/kg min^−1^): 57.84 ± 4.49 and VAT (cm): 2.87 ± 0.72) and Low V̇O_2_–High VAT (male: 72% and female: 27.78%; V̇O_2_peak (mL/kg min^−1^): 36.94 ± 6.18 and VAT (cm): 5.02 ± 1.25) (Table 1) to verify the role of cardiorespiratory fitness and VAT on global inflammatory/metabolic response and in differentiated Treg cells in the absence and presence of rapamycin and Torin 1 (mTORC 1 and 2 inhibitors). Given that body fat, age, and sex are associated with inflammatory outcomes, comparisons between experimental groups were for these covariates.

**TABLE 1:** General characteristics of young adult males and females according to cardiorespiratory fitness level and visceral adipose tissue thickness.

Variables	*N*	High V̇O_2_–Low VAT	*N*	Low V̇O_2_–High VAT	*p*‐value	*p*‐value^adj1^	*p*‐value^adj2^
*Sex*							
Male	16		13				
Female	4		5				
*Anthropometry*							
Age (y)	20	24.74 ± 4.64	18	27.69 ± 5.43	0.075	0.066	0.484
Body weight (kg)	20	69.56 ± 12.38	18	97.03 ± 29.91	**<0.001**	**<0.001**	0.781
BMI (kg/m^2^)	20	23.38 ± 2.71	18	31.72 ± 6.22	**0.001**	**0.001**	0.710
Waist circumference (cm)	20	78.94 ± 10.29	18	100.32 ± 15.50	**<0.001**	**<0.001**	0.867
VAT (cm)	20	2.87 ± 0.72	18	5.02 ± 1.25	**<0.001**	**<0.001**	**0.003**
*Physical fitness and activity*							
V̇O_2_peak (mL kg min^−1^)	20	57.84 ± 4.49	18	36.94 ± 6.18	**<0.001**	**<0.001**	**<0.001**
LPA (min/day)	16	271.72 ± 63.47	11	296.56 ± 91.19	0.411	0.466	0.331
MPA (min/day)	16	41.13 ± 27.74	11	26.84 ± 19.70	0.154	0.230	0.999
VPA (min/day)	16	9.57 ± 10.34	11	4.63 ± 10.49	0.236	0.328	0.458
MVPA (min/day)	16	50.71 ± 29.74	11	31.49 ± 25.66	0.094	0.153	0.688
SB (h/day)	16	10.65 ± 2.92	11	10.12 ± 1.60	0.574	0.656	0.783
Breaks of SB (number)	16	99.58 ± 21.47	11	90.93 ± 19.61	0.297	0.315	0.705
Length of SB (minutes)	16	9.07 ± 2.89	11	9.56 ± 4.06	0.720	**0.028**	0.496

*Note*: Data are presented as mean ± standard deviation (SD) for parametric distribution or median and interquartile range (IQR) for non‐parametric distribution. Bold value *p*‐value <0.005 and *p*‐value^adj1^ < 0.05 adjusted by sex and *p*‐value^adj2^ < 0.05 adjusted by body fat (%), BMI, waist circumference, and age.

Abbreviations: BMI, body mass index; LPA, light physical activity; MPA, moderate physical activity; MVPA, moderate to vigorous physical activity; SB, sedentary behavior; VAT, visceral adipose tissue; V̇O_2_max, maximal oxygen uptake; VPA, vigorous physical activity.

It was verified that Low V̇O_2_–High VAT young adult males and females presented higher body weight (*p*‐value = <0.001, *p*‐value adjusted^1^ = <0.001, and *p*‐value adjusted^2^ = 0.781), BMI (*p*‐value = 0.001, *p*‐value adjusted^1^ = 0.001, and *p*‐value adjusted^2^ = 0.710), waist circumference (*p*‐value = <0.001, *p*‐value adjusted^1^ = <0.001, and *p*‐value adjusted^2^ = 0.867), and length of sedentary behavior (SB) (*p*‐value = 0.720, *p*‐value adjusted^1^ = 0.028, and *p*‐value adjusted^2^ = 0.496) and lower phase angle (*p*‐value = 0.277, *p*‐value adjusted^1^ = 0.002, and *p*‐value adjusted^2^ = 0.303) compared with High V̇O_2_–Low VAT young adult males and females (Tables 1 and 2).

**TABLE 2:** Body composition in young adult males and females according to cardiorespiratory fitness level and visceral adipose tissue thickness.

Variables	*N*	High V̇O_2_–Low VAT	*N*	Low V̇O_2_–High VAT	*p*‐value	*p*‐value^adj1^	*p*‐value^adj2^
*DXA*							
BMC (g)	18	3007.22 ± 409.66	18	3049.66 ± 562.39	0.797	0.392	0.542
BMD (g.m^2^)	18	1.33 ± 0.102	18	1.35 ± 0.156	0.683	0.462	0.268
Fat‐free mass (kg)	18	55.67 ± 7.52	18	56.00 ± 14.18	0.930	0.495	**0.024**
SMI (kg.m^2^)	18	8.51 ± 1.14	18	8.95 ± 1.80	0.388	0.141	0.741
Body fat (%)	18	14.38 ± 6.23	18	34.97 ± 14.76	**<0.001**	**<0.001**	**0.002**
Android fat (%)	18	18.86 ± 10.05	18	41.44 ± 12.35	**<0.001**	**<0.001**	0.959
Gynoid fat (%)	18	20.00 ± 6.82	18	38.95 ± 9.91	**<0.001**	**<0.001**	0.207
*Ultrasound*							
SAT (cm)	20	1.11 ± 0.63	18	2.41 ± 1.02	**<0.001**	**<0.001**	0.908
VAT/SAT ratio	20	0.39 ± 0.21	18	0.50 ± 0.23	0.163	0.187	0.192
*Steatosis*							
No (%)	20	100	10	58.82	**–**	**–**	**–**
Yes (%)	0	0	8	41.17	**–**	**–**	**–**
*Steatosis grade*							
0 (%)	20	100	10	55.82	**–**	**–**	**–**
1 (%)	0	0	5	27.77	**–**	**–**	**–**
2 (%)	0	0	3	11.76	**–**	**–**	**–**
*BIA*							
Phase angle (°)	18	6.58 ± 0.98	18	6.19 ± 1.13	0.277	**0.002**	0.303

*Note*: Data are presented as mean ± standard deviation (SD) for parametric distribution or median and interquartile range (IQR) for non‐parametric distribution. Bold value *p*‐value <0.005 and *p*‐value^adj^ < 0.05 adjusted by sex and *p*‐value^adj2^ < 0.05 adjusted by body fat (%), BMI, waist circumference, and age.

Abbreviations: BIA, bioimpedance; BMC, bone mineral content; BMD, bone mineral density; DXA, dual‐energy X‐ray absorptiometry; SAT, subcutaneous adipose tissue; SMI, skeletal muscle index; VAT, visceral adipose tissue; V̇O_2_max, maximal oxygen uptake.

### 3.2 Dietary consumption, metabolic and inflammatory markers according to cardiorespiratory fitness, and VAT in young male and female

The results in Table 3 showed that Low V̇O_2_–High VAT young adult males and females presented lower total intake relative to body weight (*p*‐value = 0.065, *p*‐value adjusted^1^ = 0.035, and *p*‐value adjusted^2^ = 0.888) and lower protein intake relative to body weight (*p*‐value = 0.019, *p*‐value adjusted^1^ = 0.010, and *p*‐value adjusted^2^ = 0.546) compared with High V̇O_2_–Low VAT young males and females. Regarding the serum metabolic and inflammatory markers, the results in Table 4 showed that Low V̇O_2_–High VAT young adult males and females presented higher total cholesterol (*p*‐value = 0.002, *p*‐value adjusted^1^ = <0.001, and *p*‐value adjusted^2^ = 0.334), TAG (*p*‐value = 0.001, *p*‐value adjusted^1^ = <0.001, and *p*‐value adjusted^2^ = 0.077), glucose (*p*‐value = <0.001, *p*‐value adjusted^1^ = <0.001, and *p*‐value adjusted^2^ = <0.001), insulin (*p*‐value = 0.009, *p*‐value adjusted^1^ = 0.008, and *p*‐value adjusted^2^ = 0.168), and HOMA‐IR (*p*‐value = 0.006, *p*‐value adjusted^1^ = 0.005, and *p*‐value adjusted^2^ = 0.125) compared with High V̇O_2_–Low VAT young adult males and females.

**TABLE 3:** Dietary consumption in young adult males and females according to cardiorespiratory fitness level and visceral adipose tissue thickness.

Variables	*N*	High V̇O_2_–Low VAT	*N*	Low V̇O_2_–High VAT	*p*‐value	*p*‐value^adj1^	*p*‐value^adj2^
Total amount of food (g)	18	1492.60 ± 591.11	14	1378.97 ± 511.59	0.576	0.583	0.740
*Absolute*							
Total intake (kcal)	18	2083.92 ± 590.82	14	2074.32 ± 696.75	0.966	0.940	0.760
Carbohydrate (g)	18	254.43 ± 135.40	14	241.20 ± 130.93	0.762	0.772	0.955
Protein (g)	18	120.11 ± 54.48	14	95.62 ± 28.33	0.136	0.126	0.284
Lipid (g)	18	75.93 ± 36.15	14	81.36 ± 36.69	0.685	0.691	0.155
Polyunsaturated lipid (g)	18	15.41 ± 19.91	14	11.98 ± 3.29	0.531	0.551	0.073
Monounsaturated lipid (g)	18	24.32 ± 12.18	14	25.82 ± 9.87	0.710	0.728	0.217
Saturated lipid (g)	18	25.16 ± 10.39	14	30.15 ± 22.06	0.393	0.373	0.979
*Relative*							
Total intake (kcal/kg)	18	29.91 ± 10.26	14	23.54 ± 8.21	0.065	**0.035**	0.888
Carbohydrate (g/kg)	18	3.61 ± 2.09	14	2.78 ± 1.36	0.207	0.190	0.906
Protein (g/kg)	18	1.72 ± 0.97	14	1.06 ± 0.29	**0.019**	**0.010**	0.546
Lipid (g/kg)	18	1.58 ± 2.10	14	0.89 ± 0.34	0.233	0.242	0.529

*Note*: Data are presented as mean ± standard deviation (SD) for parametric distribution or median and interquartile range (IQR) for non‐parametric distribution. Bold value *p*‐value <0.005 and *p*‐value^adj^ < 0.05 adjusted by sex and *p*‐value^adj2^ < 0.05 adjusted by body fat (%), BMI, waist circumference, and age.

Abbreviations: V̇O_2_max, maximal oxygen uptake; VAT, visceral adipose tissue.

**TABLE 4:** Serum metabolic and inflammatory markers in young adult males and females according to cardiorespiratory fitness level and visceral adipose tissue thickness.

Variables	*N*	High V̇O_2_–Low VAT	*N*	Low V̇O_2_–High VAT	*p*‐value	*p*‐value^adj1^	*p*‐value^adj2^
*Metabolic*							
Total cholesterol (mg/dL)	20	135.43 ± 21.45	18	160.32 ± 23.87	**0.002**	**<0.001**	0.334
TAG (mg/dL)	20	102.04 ± 9.40	18	126.83 ± 24.11	**0.001**	**<0.001**	0.077
Glucose (mg/dL)	20	74.56 ± 4.55	18	84.92 ± 4.91	**<0.001**	**<0.001**	**<0.001**
Insulin (μUI/mL)	20	5.38 ± 5.41	18	15.17 ± 13.67	**0.009**	**0.008**	0.168
HOMA‐IR	20	0.69 (0.72)	18	1.92 (4.26)	**0.006**	**0.005**	0.125
*Inflammatory*							
Leptin (ηg/mL)	20	1.27 (1.45)	18	7.32 (14.93)	**<0.001**	**<0.001**	**<0.001**
Adiponectin (μg/mL)	20	4.81 ± 2.97	18	4.62 ± 2.20	0.830	0.902	0.234
Adiponectin/Leptin ratio	20	2.78 (3.58)	18	0.44 (0.48)	**0.010**	**0.011**	0.315
Leptin/VAT ratio	20	0.54 (0.89)	18	1.84 (3.06)	**0.012**	**0.010**	**0.001**
Leptin/SAT ratio	20	1.59 (3.07)	18	3.66 (11.38)	**0.023**	**0.018**	**<0.001**
Adiponectin/VAT ratio	20	1.34 (1.47)	18	0.84 (0.60)	**0.034**	**0.039**	0.986
Adiponectin/SAT ratio	20	4.20 (8.53)	17	1.76 (0.94)	**0.024**	**0.029**	0.611

*Note*: Data are presented as mean ± standard deviation (SD) for parametric distribution or median and interquartile range (IQR) for non‐parametric distribution. Bold value *p*‐value <0.005 and *p*‐value^adj^ < 0.05 adjusted by sex and *p*‐value^adj2^ < 0.05 adjusted by body fat (%), BMI, waist circumference, and age.

Abbreviations: HOMA‐IR, homeostasis model assessment‐estimated insulin resistance; SAT, subcutaneous adipose tissue; TAG, triglycerides; VAT, visceral adipose tissue; V̇O_2_max, maximal oxygen uptake.

As expected, Low V̇O_2_–High VAT young adult males and females showed higher concentrations of leptin (*p*‐value = <0.001, *p*‐value adjusted^1^ = <0.001, and *p*‐value adjusted^2^ = <0.001), leptin/VAT ratio (*p*‐value = 0.012, *p*‐value adjusted^1^ = 0.010, and *p*‐value adjusted^2^ = 0.001), leptin/SAT (*p*‐value = 0.023, *p*‐value adjusted^1^ = 0.018, and *p*‐value adjusted^2^ = 0.021), lower adiponectin/leptin ratio (*p*‐value = 0.010, *p*‐value adjusted^1^ = 0.011, and *p*‐value adjusted^2^ = 0.315), adiponectin/VAT ratio (*p*‐value = 0.034, *p*‐value adjusted^1^ = 0.039, and *p*‐value adjusted^2^ = 0.986), and adiponectin/SAT ratio (*p*‐value = 0.024, *p*‐value adjusted^1^ = 0.029, and *p*‐value adjusted^2^ = 0.611) compared with High V̇O_2_–Low VAT young adult males and females.

### 3.3 Inflammatory response in whole blood and PBMC

**FIGURE 1:**
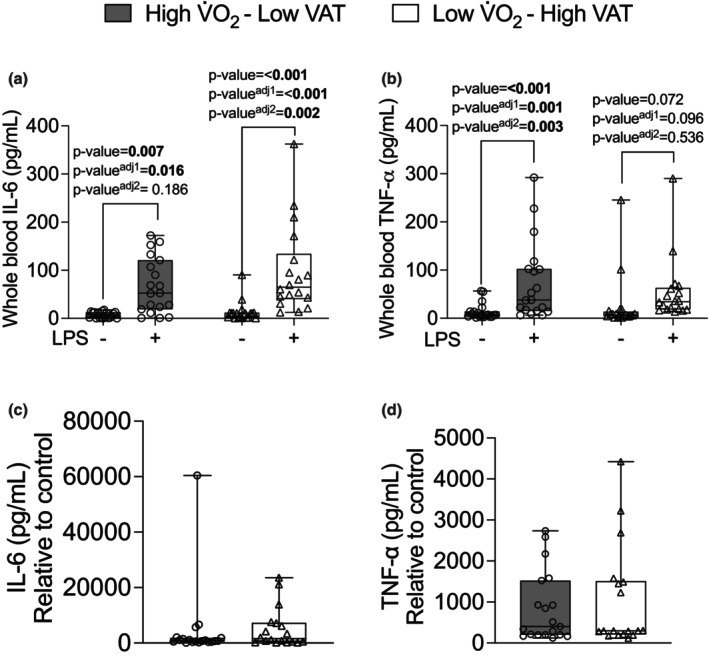
Whole blood stimulated or not with LPS (10 ng/mL). Panel (a) production of IL‐6, (b) production of TNF‐α, (c) relative change of IL‐6, and (d) relative change of TNF‐α in individuals with high V̇O_2_max–low visceral adipose tissue (VAT) (*n* = 20) and individuals with low V̇O_2_max–high VAT (*n* = 18). Data are presented as median (IQR). Bold value *p*‐value < 0.005 and *p*‐value^adj^ < 0.05 adjusted by sex and *p*‐value^adj2^ < 0.05 adjusted by body fat (%), BMI, waist circumference, and age.

To verify the role of cardiorespiratory fitness and VAT in young adult males and females blood samples were exposed to an endotoxin stimulus with LPS in a whole blood assay and LPS or PMA in PBMC culture. Low V̇O_2_–High VAT young adult males and females (*p*‐value = <0.001, *p*‐value adjusted^1^ = <0.001, and *p*‐value adjusted^2^ = 0.002) compared with the absence of LPS, as well as High V̇O_2_–Low VAT young adult males and females presented higher IL‐6 production in the presence of LPS (*p*‐value = 0.007, *p*‐value adjusted^1^ = 0.016, and *p*‐value adjusted^2^ = 0.186) compared with the absence of LPS (Figure 1a). In addition, there was a significant main effect of the production of TNF‐α in response to the stimulus with LPS in whole blood. Low V̇O_2_–High VAT young adult males and females showed a trend toward an increase of production of TNF‐α (*p*‐value = 0.072, *p*‐value adjusted^1^ = 0.096, and *p*‐value adjusted^2^ = 0.536) compared with the absence of LPS. In contrast, High V̇O_2_–Low VAT young adult males and females presented higher production of TNF‐α (*p*‐value = <0.001, *p*‐value adjusted^1^ = 0.001, and *p*‐value adjusted^2^ = 0.002) compared with the absence of LPS (Figure 1b).

**FIGURE 2:**
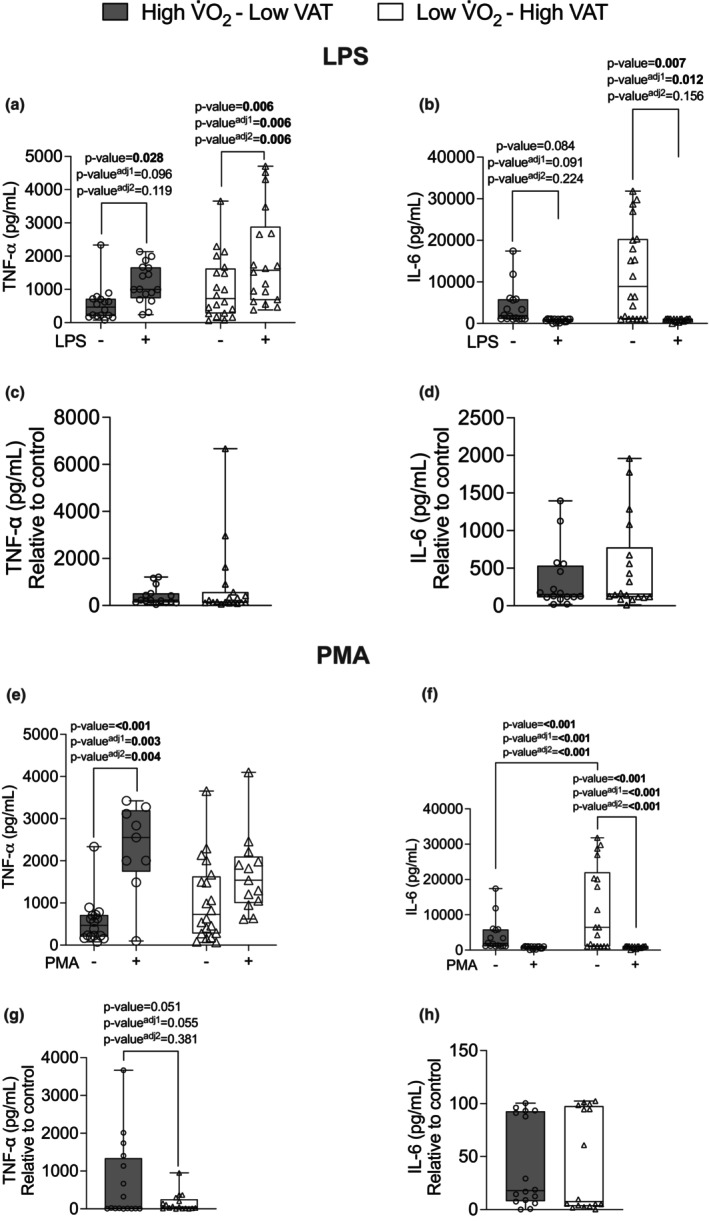
Cultured peripheral blood mononuclear cells (PBMCs) in the absence or presence of LPS (10 ng/mL). Panel (a) production of TNF‐α, (b) production of IL‐6, (c) relative change of TNF‐α, (d) relative change of IL‐6 in individuals with high V̇O_2_max–low visceral adipose tissue (VAT) (*n* = 20) and individuals with low V̇O_2_max–high VAT (*n* = 18), and in the absence or presence of PMA (50 ng/mL). (e) production of TNF‐α, (f) production of IL‐6, (g) relative change of TNF‐α, (h) relative change of IL‐6 in individuals with high V̇O_2_max–low VAT (*n* = 20) and individuals with low V̇O_2_max–high VAT (*n* = 18). Data are presented as median (IQR). Bold value *p*‐value < 0.005 and *p*‐value^adj^ <0.05 adjusted by sex and *p*‐value^adj2^ < 0.05 adjusted by body fat (%), BMI, waist circumference, and age.

In regard to the cytokine production in PBMC, there was a significant main effect on the production of TNF‐α and IL‐6 in response to stimulation with LPS. We observed that Low V̇O_2_–High VAT young adult males and females showed higher production of TNF‐α in the presence of LPS (*p*‐value = 0.006, *p*‐value adjusted^1^ = 0.006, and *p*‐value adjusted^2^ = 0.006) compared with the absence of LPS, as well as High V̇O_2_–Low VAT young adult males and females (*p*‐value = 0.028, *p*‐value adjusted^1^ = 0.096, and *p*‐value adjusted^2^ = 0.119) (Figure 2a). In addition, Low V̇O_2_–High VAT young adult males and females presented higher IL‐6 production in the presence of LPS (*p*‐value = 0.007, *p*‐value adjusted^1^ = 0.012, and *p*‐value adjusted^2^ = 0.156) compared with the absence of LPS (Figure 2b). Thus, beyond the influences of cardiorespiratory fitness and VAT, sex differences may significantly contribute to the modulation of immunomodulatory cytokine production in whole blood and LPS‐stimulated PBMC cultures.

There was further verified significant interaction main effect (group × stimulus) (*p*‐value = 0.017, *p*‐value adjusted^1^ = 0.020, *p*‐value adjusted^2^ = 0.022) in cultured PBMC with stimulus of PMA. High V̇O_2_–Low VAT young adult males and females presented higher TNF‐α production in the presence of PMA (*p*‐value = <0.001, *p*‐value adjusted^1^ = 0.003, and *p*‐value adjusted^2^ = 0.004) compared with the absence of PMA (Figure 2e). Low V̇O_2_–High VAT young adult males and females presented lower IL‐6 production in the presence of PMA (*p*‐value = <0.001, *p*‐value adjusted^1^ = <0.001, and *p*‐value adjusted^2^ = <0.001) compared with the absence of PMA (Figure 2f). Likewise, High V̇O_2_–Low VAT young adult males and females presented lower IL‐6 production in the presence of PMA (*p*‐value = <0.001, *p*‐value adjusted^1^ = <0.001, and *p*‐value adjusted^2^ = <0.001) compared with the absence of PMA (Figure 2f). Moreover, there was a trend on the production of TNF‐α relative to control; Low V̇O_2_–High VAT young adult males and females presented lower production of TNF‐α relative to control compared with High V̇O_2_–Low VAT young adult males and females (*p*‐value = 0.051, *p*‐value adjusted^1^ = 0.055, and *p*‐value adjusted^2^ = 0.381) (Figure 2g). Therefore, cultured PBMC from High V̇O_2_–Low VAT young adult males and females showed to be more responsive to stimulus with PMA and LPS for the production of TNF‐α and cultured PBMC from Low V̇O_2_– High VAT young adult males and females showed to be more responsive to stimulus with LPS for the production of TNF‐a.

### 3.4 Immunophenotyping, inhibitory immune checkpoints, CD4 ^+^ and Treg response

**FIGURE 3:**
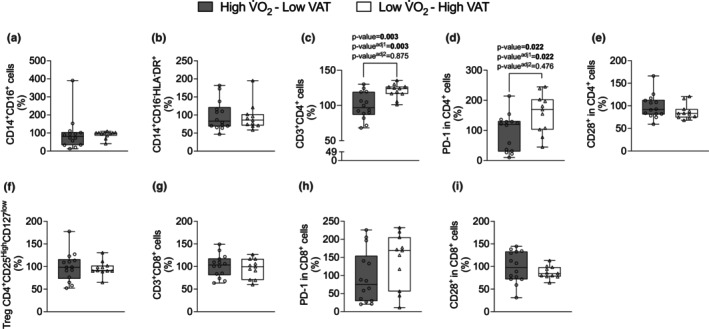
Phenotypic and checkpoint markers of peripheral blood mononuclear cell (PBMC) in individuals with high V̇O_2_max–low visceral adipose tissue (VAT) (*n* = 14) and individuals with low V̇O_2_max–high VAT (*n* = 12). Panel (a) CD14^+^CD16^+^ cells, (b) CD14^+^CD16^−^HLA^−^DR, (c) CD3^+^CD4^+^, (d) PD‐1 in CD4^+^ cells, (e) CD28^+^ in CD4^+^, (f) Treg CD4^+^CD25^high^CD127^low^ cells, (g) CD3^+^CD8^+^, (h) PD‐1 in CD8^+^ cells, (i) CD28^+^ in CD8^+^. Data are presented as mean ± SD. Bold value *p*‐value < 0.005 and *p*‐value^adj^ < 0.05 adjusted by sex and *p*‐value^adj2^ < 0.05 adjusted by body fat (%), BMI, waist circumference, and age.

**FIGURE 4:**
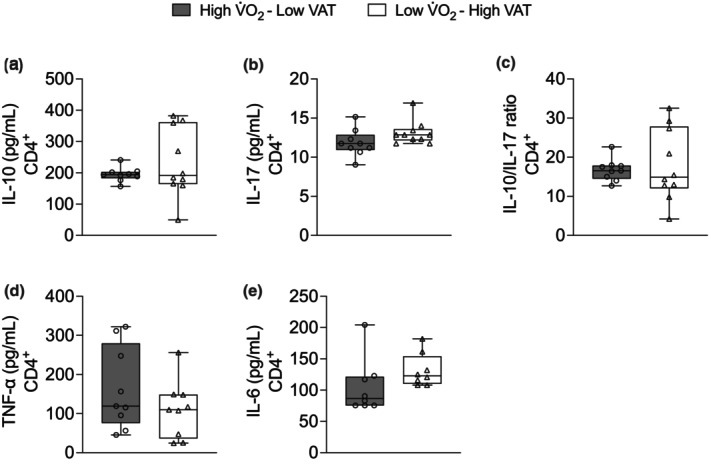
CD4^+^ isolated and cultured over 96 h without any stimulus in individuals with high V̇O_2_max–low visceral adipose tissue (VAT) (*n* = 9) and individuals with low V̇O_2_max–high VAT (*n* = 10). Panel (a) production of IL‐10, (b) production of IL‐17, (c) IL‐10/IL‐17 ratio, (d) production of TNF‐α, and (e) production of IL‐6. Data are presented as mean ± SD. Bold values represent *p*‐value < 0.05 adjusted by sex and *p*‐value^adj2^ < 0.05 adjusted by body fat (%), BMI, waist circumference, and age.

**FIGURE 5:**
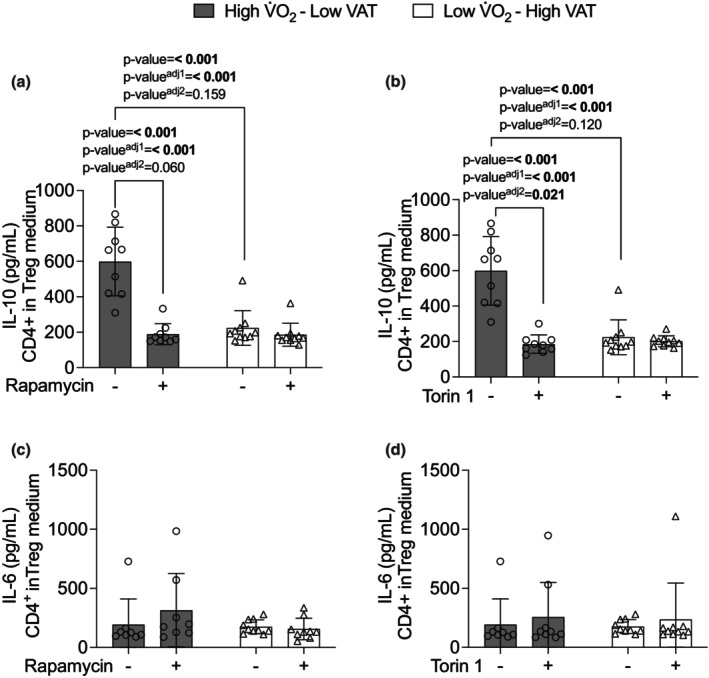
CD4^+^ differentiated in Treg in presence of 100 ηM of rapamycin (mTORC1) and 50 ηM of Torin‐1 (mTORC2) in individuals with high V̇O2max–low visceral adipose tissue (VAT) (*n* = 9) and individuals with low V̇O2max–high VAT (*n* = 10). Panel (a) production of IL‐10 with 100 ηM of rapamycin (mTORC1), (b) production of IL‐10 with 50 ηM of Torin‐1 (mTORC2), (c) production of IL‐6 with 100 ηM of rapamycin (mTORC1), (d) production of IL‐6 with 50 ηM of Torin‐1 (mTORC2). Data are presented as mean ± SD. Bold values represent *p*‐value < 0.05 adjusted by sex and *p*‐value^adj2^ < 0.05 adjusted by body fat (%), BMI, waist circumference, and age.

The results of Figure 3 showed that Low V̇O_2_–High VAT young adult males and females presented a higher frequency of CD4^+^ (*p*‐value = 0.003, *p*‐value adjusted^1^ = 0.003, and *p*‐value adjusted^2^ = 0.875) (Figure 3c), and frequency of PD‐1 in CD4^+^ (*p*‐value = 0.022, *p*‐value adjusted^1^ = 0.022, and *p*‐value adjusted^2^ = 0.476) compared with High V̇O_2_–Low VAT young adult males and females (Figure 3d). We next isolated CD4^+^ from whole blood of young adult males and females and cultured over 96 h in absence of stimulus. No significant changes were observed in cytokine production (Figure 4). It was further isolated CD4^+^ to Treg differentiation in presence of 2 ng/mL of TGF‐β over 96 h in absence or presence of 100 nM of rapamycin or 50 nM of Torin‐1. We observed that Low V̇O_2_–High VAT young adult males and females showed low production of IL‐10 from Tregs in absence of rapamycin compared with High V̇O_2_–Low VAT young adult males and females Tregs in absence of rapamycin (*p*‐value = <0.001, *p*‐value adjusted^1^ = <0.001, and *p*‐value adjusted^2^ = 0.159) (Figure 5a). In addition, High V̇O_2_–Low VAT young adult males and females showed lower IL‐10 production from Tregs in presence of rapamycin compared with absence of rapamycin (*p*‐value = <0.001, *p*‐value adjusted^1^ = <0.001, and *p*‐value adjusted^2^ = 0.060) (Figure 5a). In regard to Torin‐1 stimulus, similarly we observed that Low V̇O_2_–High VAT young adult males and females showed low production of IL‐10 from Tregs in absence of Torin‐1 compared with High V̇O_2_–Low VAT young adult males and females Tregs in absence of Torin‐1 (*p*‐value = <0.001, *p*‐value adjusted^1^ = <0.001, and *p*‐value adjusted^2^ = 0.120) (Figure 5b). High V̇O_2_–Low VAT young adult males and females presented lower IL‐10 production from Tregs in presence of Torin‐1 (*p*‐value = <0.001, *p*‐value adjusted^1^ = <0.001, and *p*‐value adjusted^2^ = 0.021) compared with absence of Torin‐1 (Figure 5b).

### 3.5 Correlation of V̇O_2_peak and VAT

**FIGURE 6:**
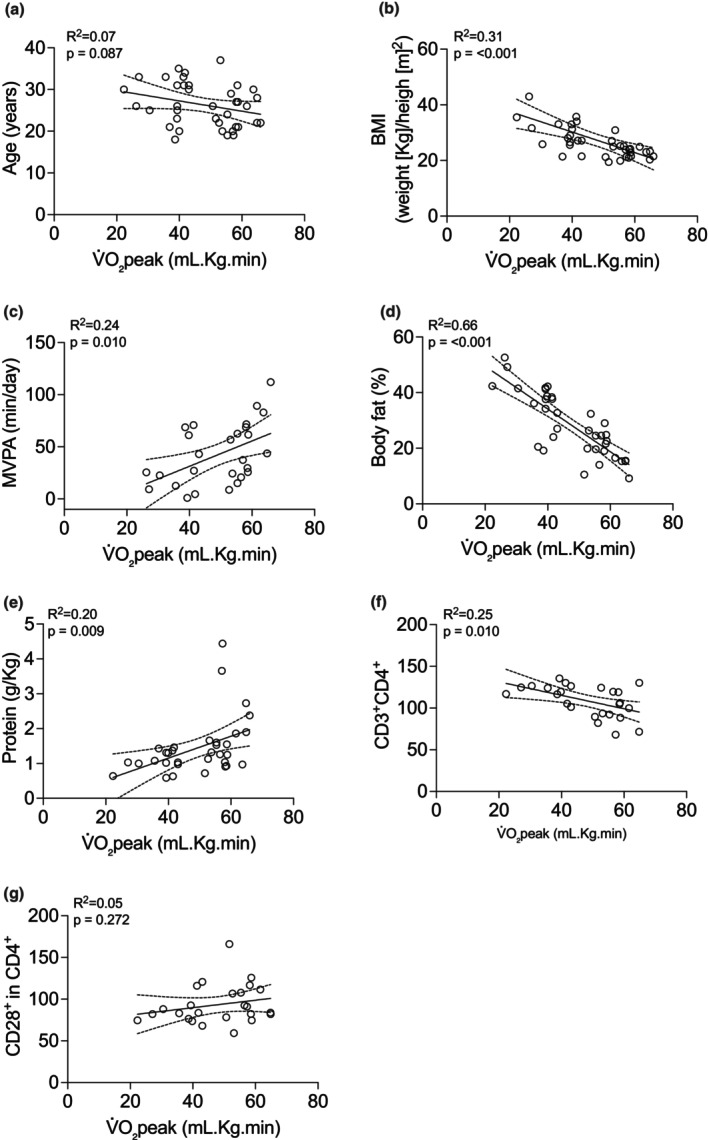
Pearson's exploratory correlations of V̇O_2_peak in individuals with high V̇O_2_peak–low VAT (*n* = 9) and individuals with low V̇O_2_peak–high VAT (*n* = 10). Panel (a) V̇O_2_peak with age, (b) V̇O_2_peak with BMI, (c) V̇O_2_peak with MVPA, (d) V̇O_2_peak with percentage of body fat, (e) V̇O_2_peak with protein consumption, (f) V̇O_2_peak with CD3^+^CD4^+^, (g) V̇O_2_peak with CD28^+^ expression in CD4^+^.

**FIGURE 7:**
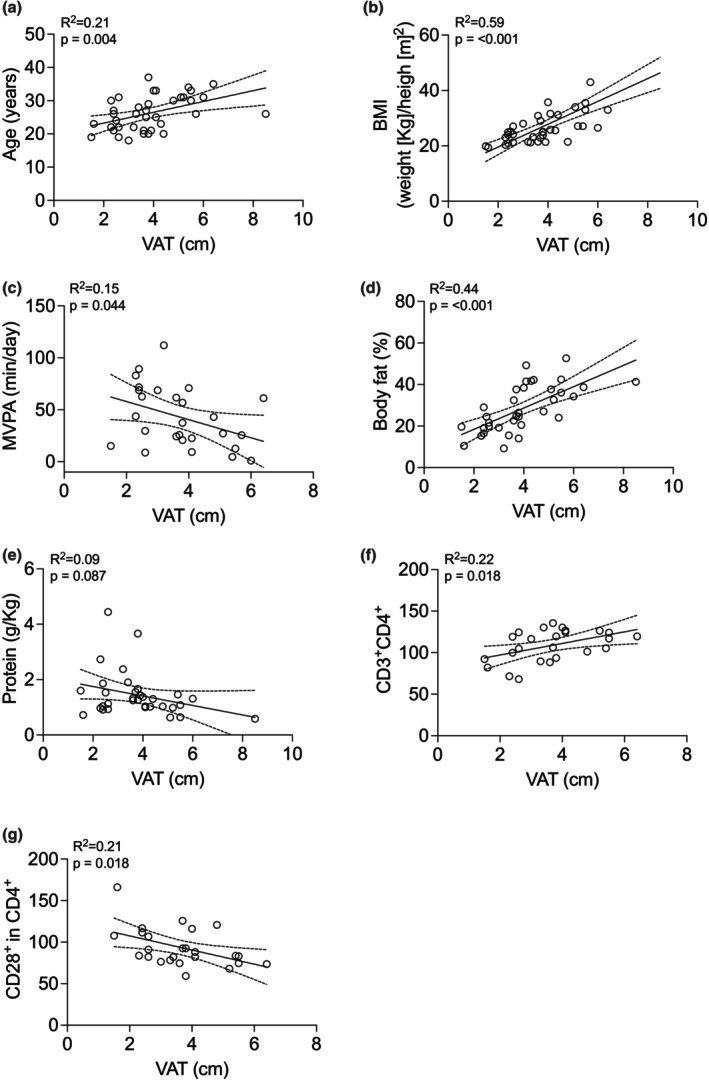
Pearson's exploratory correlations of VAT in individuals with high V̇O_2_peak–low VAT (*n* = 9) and individuals with low V̇O_2_peak–high VAT (*n* = 10). Panel (a) VAT with age, (b) VAT with BMI, (c) VAT with MVPA, (d) VAT with percentage of body fat, (e) VAT with protein consumption, (f) VAT with CD3^+^CD4^+^, (g) VAT with CD28^+^ expression in CD4^+^.

Exploratory correlation analyses revealed that V̇O₂peak was significantly associated with several physiological and immunological parameters. Specifically, higher V̇O₂peak correlated with lower BMI (*r* ^2^ = 0.31, *p* < 0.001) (Figure 6b), greater MVPA (*r* ^2^ = 0.24, *p* = 0.010) (Figure 6c), lower body fat percentage (*r* ^2^ = 0.66, *p* < 0.001) (Figure 6d), higher protein intake (*r* ^2^ = 0.20, *p* = 0.009) (Figure 6e), and a higher frequency of CD3^+^CD4^+^ T cells (*r* ^2^ = 0.25, *p* = 0.010) (Figure 6f). Additionally, VAT was positively correlated with age (*r* ^2^ = 0.21, *p* = 0.004) (Figure 7a), BMI (*r* ^2^ = 0.59, *p* < 0.001) (Figure 7b), body fat percentage (*r* ^2^ = 0.44, *p* < 0.001) (Figure 7d), and CD3^+^CD4^+^ T cells (*r* ^2^ = 0.22, *p* = 0.018) (Figure 7f), while showing a negative correlation with MVPA (*r* ^2^ = 0.15, *p* = 0.044) (Figure 7c). Moreover, VAT was positively associated with CD28 expression in CD4^+^ T cells (*r* ^2^ = 0.21, *p* = 0.018) (Figure 7g), suggesting a potential link between central adiposity and altered T‐cell phenotypes.

## 4 DISCUSSION

This study presents an in‐depth analysis to verify the impact of cardiorespiratory fitness and central obesity (VAT) on systemic metabolic and inflammatory status, the inflammatory response of whole blood cells and PBMC to in vitro stimulation with two immunogens, the inflammatory and stimulatory phenotype of PBMC, and CD4^+^ T cells inflammatory response before and after differentiation into Treg cells associated with mTORC1 and mTORC2 inhibition in young adult males and females. The main and novel findings of this study were that the inhibition of mTORC1 and mTORC2 blunted IL‐10 release in Treg cells from high but not low cardiorespiratory fitness individuals. In addition, beyond cardiorespiratory fitness status and central obesity (VAT), sex appears to be a determinant for the length of sedentary behavior and phase angle, relative total intake, and protein intake. Furthermore, sex appears to be a determinant for TNF‐α production in response to LPS stimulation in Low V̇O_2_–High VAT young adult males and females' cultured PBMC. These findings are significant because they highlight the sex‐dependent difference in immune system modulation according to cardiorespiratory fitness and central obesity (VAT).

Sex is a fundamental biological variable that influences both immune system function and adipose tissue distribution (Klein & Flanagan, 2016; Porter et al., 2020). Typically, males present with greater VAT accumulation—commonly referred to as an android fat distribution—whereas females tend to accumulate more SAT, following a gynoid pattern (Barsky & Monks, 2025; Borbélyová et al., 2021). Estrogen receptors (ERs) are highly expressed in adipose tissue, and estrogen signaling has been shown to regulate fat partitioning, enhance insulin sensitivity, and attenuate pro‐inflammatory signaling. Concurrently, there is substantial evidence indicating that testosterone plays a critical role in the regulation of energy metabolism and the structural maintenance of both skeletal muscle and adipose tissue (Bailey & Ahmed‐Sorour, 1980; Porter et al., 2020; Ramamani et al., 1999; van Nas et al., 2009). For instance, estrogen has been shown to enhance type I interferon signaling in female monocytes and plasmacytoid dendritic cells, contributing to heightened antiviral responses (Gubbels Bupp et al., 2018). Similarly, T lymphocytes from females often exhibit increased proliferative capacity and cytokine production compared with males, which has been linked to differential T‐cell receptor signaling and mitochondrial metabolism (Klein & Flanagan, 2016; Taneja, 2018). Collectively, these differences contribute to the observed pattern wherein females typically generate more robust immune responses to pathogens but are also more prone to developing autoimmune diseases. Conversely, males may experience greater morbidity during infections yet show lower overall prevalence of autoimmune diseases (Barsky & Monks, 2025; Gupta et al., 2020; Klein & Flanagan, 2016; Porter et al., 2020).

Our results align with previous studies showing that individuals with low cardiorespiratory fitness and high VAT display a pro‐inflammatory PBMC profile, marked by increased LPS‐stimulated IL‐6 and TNF‐α secretion (Dorneles et al., 2021; Olean‐Oliveira et al., 2022, 2023; Rosa‐Neto et al., 2022). This may reflect greater NF‐κB activation and epigenetic changes, such as histone H4 acetylation, that amplify inflammatory responses (De Loera‐Rodriguez et al., 2014). Notably, including sex as a covariate reduced these associations, suggesting sex‐specific modulation of inflammatory markers. Energy balance and macronutrient composition are well‐established modulators of immune function and systemic inflammation (Pearce, 2010; Powell et al., 2012). Higher protein intake, particularly in lean, fit individuals, was associated with anti‐inflammatory effects, likely due to improved nutrient sensing and immune cell function (Calder, 2020; Li et al., 2007). Our findings underscore the importance of considering sex differences in energy metabolism when assessing dietary influences on immune regulation.

Cytokine secretion patterns are closely influenced by the phenotypic characteristics of circulating immune cells. Furthermore, we observed that individuals with low V̇O₂ and high VAT exhibited higher proportions of CD4^+^ T cells expressing PD‐1, suggesting T‐cell exhaustion (Eljaafari et al., 2021; Wang et al., 2019). These individuals also had reduced frequencies of classical monocytes and increased leptin/VAT ratios—potential drivers of chronic low‐grade inflammation (Dorneles et al., 2021; Schwartz et al., 2022). PD‐1 overexpression may impair Treg differentiation by interfering with IL‐2/TGF‐β–driven pathways, particularly FoxP3 expression, further limiting resolution of inflammation (Ai et al., 2020).

A plausible mechanism underlying immune cell exhaustion and impaired inflammatory responses is leptin secretion, which plays a central role in modulating monocyte and lymphocyte activity, including cytokine production (TNF‐α, IL‐6, and IL‐1) (Conde et al., 2010). In our study, individuals with low V̇O₂ and high VAT showed elevated leptin levels and leptin/VAT ratios compared to those with high V̇O₂ and low VAT, coinciding with increased TNF‐α secretion. These findings suggest that chronic hyperleptinemia in low‐fit, centrally obese individuals may promote cytokine overproduction and immune cell exhaustion. Prior research links low cardiorespiratory fitness to a higher risk of cardiovascular disease and cancer (Ross et al., 2016), and even among individuals with similar VAT, those with greater aerobic fitness tend to exhibit more favorable inflammatory and metabolic profiles (Wedell‐Neergaard et al., 2018). This supports the idea that fitness mitigates the harmful effects of VAT by enhancing mitochondrial metabolism, reducing oxidative stress, and supporting immune function (Handschin & Spiegelman, 2008; Ross et al., 2016).

In this study, the highest IL‐10 production was observed in control Treg cultures (without inhibitors), particularly among individuals with high V̇O₂peak and low VAT. This suggests that greater cardiorespiratory fitness is associated with enhanced IL‐10 secretion during Treg differentiation, reflecting improved immune regulatory capacity. In contrast, the reduced IL‐10 release in individuals with low V̇O₂peak and high VAT may indicate a deficiency in immune regulation that appears independent of sex. This IL‐10–producing phenotype was abolished by mTORC1 (rapamycin) and mTORC2 (Torin‐1) inhibition, highlighting the essential role of both pathways in maintaining Treg function. IL‐6 production remained unchanged by mTOR inhibition, regardless of fitness or adiposity. These findings suggest that impaired Treg responses in low‐fit, high VAT individuals may result from immune exhaustion or metabolic dysfunction (Dommel & Blüher, 2021; Kwiat et al., 2022; Taylor, 2021). A limitation is the absence of additional stimulation (e.g., PMA/ionomycin) to confirm Treg function. Recent data (Gebhardt et al., 2024) show that higher fitness promotes oxidative phosphorylation in CD4^+^ cells, supporting anti‐inflammatory function. Collectively, our findings highlight fitness as a potential modulator of immune regulation, with implications for chronic inflammation in obesity‐related disease.

This study had some limitations. First, separate analysis of male and female subjects was not possible due to the underpowered female group. This was due to unforeseen circumstances during the COVID‐19 pandemic; several female participants from the initial screening cohort either tested positive for anti‐SARS‐CoV‐2 antibodies (IgG and IgM) or began using contraceptive methods. Second, stratifying individuals showing high VAT into low and high V̇O_2_, as well as individuals showing low VAT into low and high V̇O_2_, would allow to explore the contribution of VAT as an independent outcome. Third, while CD4^+^ cells were cultured under standard Treg differentiation conditions, we acknowledge that confirmatory assays—such as FoxP3 expression analysis or functional suppression assays—would be decisive to verify the successful differentiation into Treg cells. However, the findings shown in this study encourage future investigation involving male and female participants.

In conclusion, low cardiorespiratory fitness and high visceral adiposity play a crucial role in the inflammatory response of peripheral immune cells, especially regarding the effect on exhaustion markers in CD4^+^ and lower release of IL‐10 after differentiation of these cells into their regulatory phenotype. Also, mTORC 1 and mTORC2 appear to mediate primarily the IL‐10 release in Treg cells from high but not low cardiorespiratory fitness individuals.

## AUTHOR CONTRIBUTIONS

CSP and FSL conceived the study. CSP, TOO, CF, VRS, GPD, and JPJR collected the data. CSP, TOO, CF, VRS, GPD, and JPJR analyzed and interpreted the data. CSP, TOO, CF, VRS, GPD, JPJR, RD, KK, JCR, and FSL wrote the first draft. All authors reviewed the first draft and approved the final version submitted and published version.

## FUNDING INFORMATION

We thank the São Paulo Research Foundation‐Brazil (FAPESP) and Coordenação de Aperfeiçoamento Pessoal de Nível Superior Brazil (CAPES) #001. CSP has been granted a Post Doctorate fellowship from FAPESP (Process 2018/23402‐0), TOO has been granted a Master scholarship from FAPESP (Process 2019/11340‐3), CF has been granted a doctorate scholarship (Process 2019/26378‐6), and FSL has been granted research grants from FAPESP (Process 2018/19678‐0; 2022/13978‐8; 2024/02320‐7).

## CONFLICT OF INTEREST STATEMENT

The authors declare no conflict of interest.

## ETHICS STATEMENT

This study was approved by the local Research Ethics Committee of the São Paulo State University “Júlio de Mesquita Filho” and duly registered in the Brazil Platform.
