# Correction to “CD4+ differentiated T regulatory cells is modified by physical fitness and visceral adipose tissue in young adults—A cross‐sectional study”

**DOI:** 10.14814/phy2.70839

**Published:** 2026-04-15

Padilha, C. S., Olean‐Oliveira, T., Figueiredo, C., Dos Santos, V. R., Dorneles, G. P., Ribeiro, J. P. J., Deminice, R., Krüger, K., Rosa‐Neto, J. C., Lira, F. S. CD4+ differentiated T regulatory cells is modified by physical fitness and visceral adipose tissue in young adults‐A cross‐sectional study. *Physiological Reports*, 2025, 13(16), e70470. https://doi.org/10.14814/phy2.70470.

The authors regret an error in the published version of Figure 2. Panel (b) of Figure 2, which shows IL‐6 production in cultured peripheral blood mononuclear cells stimulated with LPS, was incorrect. The bars for the +LPS conditions were incorrect and inadvertently replicated the +LPS bars presented in Fig. 2(f).

The updated Figure 2 with the correct panel 2(b) is provided below.

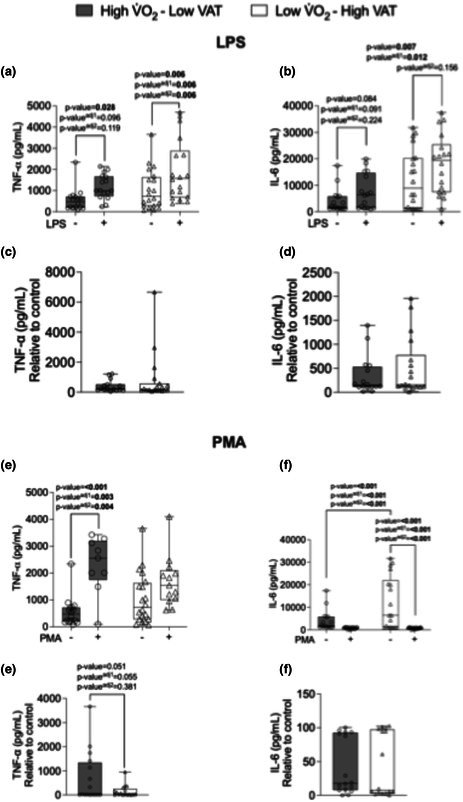

We apologize for this error, which does not affect the scientific conclusions of the study.

